# A high α1-antitrypsin/interleukin-10 ratio predicts bacterial pneumonia in adults with community-acquired pneumonia: a prospective cohort study

**DOI:** 10.1186/s41479-023-00118-4

**Published:** 2023-10-25

**Authors:** Taiga Miyazaki, Kiyoyasu Fukushima, Kohji Hashiguchi, Shotaro Ide, Tsutomu Kobayashi, Toyomitsu Sawai, Kazuhiro Yatera, Yoshihisa Kohno, Yuichi Fukuda, Yoji Futsuki, Yuichi Matsubara, Hironobu Koga, Tomo Mihara, Eisuke Sasaki, Nobuyuki Ashizawa, Tatsuro Hirayama, Takahiro Takazono, Kazuko Yamamoto, Yoshifumi Imamura, Norihito Kaku, Kosuke Kosai, Yoshitomo Morinaga, Katsunori Yanagihara, Hiroshi Mukae

**Affiliations:** 1 Division of Respirology, Rheumatology, Infectious Diseases, and Neurology, Department of Internal Medicine, Faculty of Medicine, University of Miyazaki, Miyazaki, Japan; 2 Department of Respiratory Medicine, Nagasaki University Hospital, Nagasaki, Japan; 3 Japanese Red Cross Nagasaki Genbaku Isahaya Hospital, Isahaya, Japan; 4 Japanese Red Cross Nagasaki Genbaku Hospital, Nagasaki, Japan; 5 Isahaya General Hospital, Isahaya, Japan; 6 Sasebo Chuo Hospital, Sasebo, Japan; 7 Nagasaki Harbor Medical Center, Nagasaki, Japan; 8 Department of Respiratory Medicine, University of Occupational and Environmental Health, Japan, Kitakyushu, Japan; 9 Kouseikai Hospital, Nagasaki, Japan; 10 Sasebo City General Hospital, Sasebo, Japan; 11 Saiseikai Nagasaki Hospital, Nagasaki, Japan; 12 Juko Memorial Nagasaki Hospital, Nagasaki, Japan; 13 Aino Memorial Hospital, Unzen, Japan; 14 Nagasaki Medical Center, Omura, Japan; 15 Ureshino Medical Center, Ureshino, Japan; 16 Department of Pharmacotherapeutics, Nagasaki University Graduate School of Biomedical Science, Nagasaki, Japan; 17 First Department of Internal Medicine, Division of Infectious, Respiratory, and Digestive Medicine, University of the Ryukyus Graduate School of Medicine, Okinawa, Japan; 18 Medical Education Development Center, Nagasaki University Hospital, Nagasaki, Japan; 19 Department of Laboratory Medicine, Nagasaki University Graduate School of Biomedical Sciences, Nagasaki, Japan; 20 Department of Microbiology, Graduate School of Medicine and Pharmaceutical Sciences, University of Toyama, Toyama, Japan

**Keywords:** Community-acquired pneumonia, Biomarker, Interleukin-10, α1-antitrypsin, Bacterial pneumonia

## Abstract

**Background:**

Current microbiological tests fail to identify the causative microorganism in more than half of all pneumonia cases. We explored biomarkers that could be used for differentiating between bacterial and viral pneumonia in patients with community-acquired pneumonia (CAP).

**Methods:**

In this prospective cohort study conducted in Japan, data obtained from adult patients with bacterial pneumonia, including bacterial and viral coinfections (bacterial pneumonia [BP] group), and purely viral pneumonia (VP group) at diagnosis were analyzed using multivariate logistic regression analysis to identify predictors of bacterial pneumonia. Furthermore, a decision tree was developed using the predictors.

**Results:**

A total of 210 patients were analyzed. The BP and VP groups comprised 108 and 18 patients, respectively. The other 84 patients had no identified causative microorganism. The two groups shared similar characteristics, including disease severity; however, a significant difference (*p* < 0.05) was observed between the two groups regarding sputum type; sputum volume score; neutrophil counts; and serum levels of interleukin (IL)-8, IL-10, and α1-antitrypsin (AAT). Sputum volume score (*p* < 0.001), IL-10 (*p* < 0.001), and AAT (*p* = 0.008) were ultimately identified as predictors of BP. The area under the curve for these three variables on the receiver operating characteristic (ROC) curve was 0.927 (95% confidence interval [CI]: 0.881–0.974). The ROC curve for sputum volume score and an AAT/IL-10 ratio showed a diagnostic cutoff of 1 + and 65, respectively. Logistic regression analysis using dichotomized variables at the cutoff values showed that the odds ratios for the diagnosis of BP were 10.4 (95% CI: 2.2–50.2) for sputum volume score (absence vs. presence) and 19.8 (95% CI: 4.7–83.2) for AAT/IL-10 ratio (< 65 vs. ≥ 65).

**Conclusions:**

Considering that obtaining a definitive etiologic diagnosis with the current testing methods is difficult and time consuming, a decision tree with two predictors, namely sputum volume and the AAT/IL-10 ratio, can be useful in predicting BP among patients diagnosed with CAP and facilitating the appropriate use of antibiotics.

**Trial registration:**

UMIN000034673 registered on November 29, 2018.

**Supplementary Information:**

The online version contains supplementary material available at 10.1186/s41479-023-00118-4.

## Background

The causative microorganisms of community-acquired pneumonia (CAP) include a wide variety of bacteria, viruses, and atypical pathogens, which remain difficult to quickly and accurately identify with current diagnostic techniques [1–3]. Early initiation of appropriate antimicrobial agents for bacterial pneumonia is necessary to improve prognosis. In daily clinical practice, antimicrobial therapy is initiated mainly based on symptoms (e.g., presence of purulent sputum), hematological examination (e.g., neutrophilic leukocytosis), chest imaging (e.g., pulmonary infiltrative shadow), and sputum Gram staining, with comprehensive consideration of disease severity. Nonetheless, avoiding the unnecessary use of antimicrobial agents is also important to prevent the increase in antimicrobial resistance, adverse drug reactions, and healthcare costs. However, accurately identifying pneumonia that does not require antimicrobial therapy at the time of initial diagnosis remains challenging given the lack of reliable diagnostic tests. In addition, throughout the coronavirus disease 2019 (COVID-19) pandemic period, microbiological testing using respiratory specimens had been omitted in a considerable number of cases. Therefore, research on biomarkers useful in differentiating bacterial from viral pneumonia and in determining the need for antimicrobial administration has attracted much attention. One example of such biomarkers is procalcitonin, for which positive and negative findings have been found through many clinical studies [4–8]. At present, the American Thoracic Society and Infectious Diseases Society of America (ATS/IDSA) guideline [9] does not recommend the use of serum procalcitonin levels to discriminate between bacterial and viral pneumonia (as a criterion for antimicrobial use) considering the difficulty of setting an appropriate cutoff value.

Given that current microbiological tests fail to identify the causative microorganism in more than half of all pneumonia cases [10–12], an adjunctive diagnostic approach with biomarkers may facilitate the appropriate use of antimicrobial agents. In addition to some common biomarkers for sepsis and inflammation, including C-reactive protein (CRP), procalcitonin, presepsin, interleukin (IL)-6, IL-8, and IL-10, α1-antitrypsin (AAT) and pentraxin 3 were examined in the current study. AAT is a major liver-derived circulating protein that inhibits neutrophil elastase in the lungs, and deficiency of AAT has been associated with early onset emphysema [13]. AAT is also produced in alveolar epithelial cells [14], neutrophils, monocytes, and macrophages [15] and exhibits anti-inflammatory effects on peripheral blood mononuclear cells and functions as an endogenous inhibitor of proinflammatory cytokine production in whole blood [16]. Although the previous study found that AAT level was increased in pediatric patients with bacterial pneumonia compared to those with viral pneumonia [17], its utility in adult patients with CAP remains to be studied. Pentraxin 3 is a soluble pattern recognition molecule secreted by various cell types, including dendric cells, monocytes, and macrophages, in response to proinflammatory cytokines and microbial recognition [18]. Pentraxin 3 acts as an acute-phase response protein that modulates innate immunity against fungal, bacterial, and viral pathogens. However, association of this biomarker with etiology of CAP is less clear. In the current study, we conducted a multicenter prospective clinical study of patients with CAP to explore biomarkers useful in differentiating bacterial from viral pneumonia.

## Methods

### Study design

This was an open, multicenter, non-interventional, prospective cohort study conducted in Japan. We collected information on study subjects who visited our institute for the diagnosis and treatment of pneumonia after obtaining the written informed consent. During routine medical care, biomarkers were measured using blood samples, and causative pathogens were identified.

This study was registered in the UMIN Clinical Trials Registry (ID: UMIN000034673) before study initiation (November 29, 2018), was approved by the Institutional Review Board or Ethics Committee for each site, and was performed in accordance with the Declaration of Helsinki revised in October 2013 and the Japanese Ethical Guidelines for Medical and Biological Research Involving Human Subjects partially amended on February 28, 2017. The current study enrolled patients who visited 14 institutions throughout Kyushu, Japan between December 2018 and December 31, 2020.

### Study subjects

Eligible patients were adults aged ≥ 20 years who were diagnosed with CAP in accordance with the ATS/IDSA guideline or Japanese guidelines for the treatment of adult pneumonia and had satisfied the following two conditions:

1. Two or more of the following findings/symptoms associated with pneumonia: cough (productive or dry); purulent sputum; abnormal findings on auscultation or percussion (e.g., moist rales, diminished breath sounds, abnormal turbidity on percussion); dyspnea or tachypnea; fever (axillary body temperature ≥ 37 °C); increased white blood cells (WBCs) (> 10,000/mm^3^), increased stab cells (> 15%), or decreased WBCs (< 4,500/mm^3^); elevated CRP levels (> upper limit of normal at each institution); and hypoxemia (PaO_2_ < 60 Torr or SpO_2_ < 90%).
2. Findings suspicious for pneumonia (e.g., alveolar infiltration shadows on air bronchograms, pleural effusion, or other new increased lung shadows suspicious for infection) on chest radiographs or chest computed tomography images obtained within 48 h of subject registration.

Exclusion criteria included pneumonia that occurred after 48 h of hospitalization; patients who had been enrolled in this study; those who had already started antimicrobial therapy for this episode (pneumonia) and had shown improvement (patients who had received antimicrobial therapy, in principle, for a minimum of 3 days and had not shown improvement were allowed entry; however, this rule was applied only if the investigators determined that the patient was appropriate as a study subject); those with respiratory infections caused by *Mycoplasma pneumoniae*, *Chlamydophila pneumoniae*, *Bordetella pertussis*, *Pneumocystis jirovecii*, or mycobacterial species (including suspected cases); those who had used azithromycin within 7 days before study treatment initiation, excluding low-dose macrolide regimens (low-dose macrolide therapies before study treatment initiation were allowed to continue without changes in the dosage); those with an underlying disease that can significantly impact the diagnosis of CAP in this study, such as advanced cancer, primary lung cancer, lung metastasis of malignant tumors, severe heart failure, cystic fibrosis, and acquired immunodeficiency syndrome; and those determined to be inappropriate as study subjects by the investigators.

### Data collection

Data obtained from patients and medical records included age, sex, height, weight, inpatient/outpatient status, underlying diseases, symptoms (temperature, cough, and sputum), disease severity [Acute Physiology and Chronic Health Evaluation II (APACHE-II) score, Confusion, Urea, Respiratory Rate, Blood Pressure and Age ≥ 65 Years (CURB-65) score, and Pneumonia Severity Index (PSI)], blood biomarkers, treatment drug, and safety information.

If possible, sputum was collected before antimicrobial administration (Day 0), and Gram staining and culture tests were performed. Sputum volume score [5 steps: 0 (none), 1 + (< 10 mL/day), 2 + (10 to < 50 mL/day), 3 + (50 to < 100 mL/day), 4 + (≥ 100 mL/day)] and sputum purulence [purulent (P), purulent-mucous (PM), and mucous (M)] [19] were also recorded. The causative microorganisms were identified based on data from samples obtained on Day 0 and subsequent days if necessary using sputum culture, urine antigen tests (*Streptococcus pneumoniae* and *Legionella pneumophila*), sputum antigen tests (*S. pneumoniae)*, and FilmArray assay of nasopharyngeal swabs. The FilmArray respiratory panel can detect 20 pathogens including viruses [Adenovirus, Coronavirus (229E, HKU1, OC43, NL63), Human Metapneumovirus, Human Rhinovirus/Enterovirus, Influenza A (A/H1, A/H1-2009, A/H3), Influenza B, Parainfluenza 1–4, Respiratory Syncytial Virus] and atypical organisms (*M. pneumoniae*, *C. pneumoniae*, and *B. pertussis*). Assays were conducted by laboratory technicians at Department of Laboratory Medicine, Nagasaki University Hospital (Nagasaki, Japan) who were blinded to the patient information.

Blood samples were obtained on Day 0 for routine blood laboratory tests and biomarker measurements. Routine blood laboratory tests including blood cell counts, random plasma glucose, hemoglobin A1c (HbA1c), aspartate aminotransferase, alanine aminotransferase, alkaline phosphatase (ALP), γ-glutamyl transpeptidase, lactate dehydrogenase, total bilirubin, blood urea nitrogen, creatinine, and CRP were performed at participating hospitals. For additional biomarker measurements, serum (for IL-6, IL-8, IL-10, AAT, procalcitonin, and presepsin) and plasma (for pentraxin 3) were separated and cryopreserved immediately after blood collection. These biomarkers were measured at LSI Medience Corporation (Tokyo, Japan).

### Data analyses

All cases were first classified into the following four categories based on the identified causative microorganism according to the flow diagram in Fig. S1: bacteria alone (purely bacterial pneumonia), virus alone (purely viral pneumonia), bacterial and viral coinfection (mixed bacterial–viral pneumonia), and none (no organism). Background data were presented as number (%) or median (interquartile).

The normality of continuous variables, including age, disease severity (PSI and CURB-65 score), sputum volume score, blood cell numbers (neutrophils, lymphocytes, and platelets), and biomarkers, was assessed using the Kolmogorov–Smirnov test, and variables were normalized using the Box-Cox transformation. Tests for outliers were performed with the Smirnov–Grubbs test. However, all outliers excluding abnormal data were used for analysis. Given that three sputum types were available for selection (P, PM, and M), some investigators selected M or blank in cases with no sputum or low sputum volume scores, which can indicate saliva. Therefore, sputum specimens were categorized into two types, namely P/PM and M/blank.

To identify items specific to bacterial pneumonia and unpredictable background effect on diagnosis, two data sets were prepared, one for the group of patients with purely bacterial pneumonia or mixed bacterial–viral pneumonia (BP group) and another for the group of those with purely viral pneumonia (VP group). Variables, including age, sex, disease severity, biomarkers, blood cell count, and sputum type, were then compared between the two groups. Continuous variables were compared using Mann–Whitney U test, whereas binary variables were compared using Fisher’s exact test. In addition, multivariate logistic regression analysis was performed with diagnosis (BP or VP) as the objective variable and age, sex, biomarkers, blood cell numbers, and sputum type and volume as explanatory variable, adjusting for age and sex as confounding factors. Furthermore, multivariate logistic regression analysis was repeated with the diagnosis (BP or VP) as the objective variable and items with a *p* value of < 0.05 in the previous univariate or multivariate logistic regression analysis, as well as age and sex, as explanatory variables while excluding items that were not significant (*p* ≥ 0.05). However, age and sex were retained as confounding factors until the end of the analysis.

The cutoff value for the identified items was determined using the receiver operating characteristic (ROC) curve and Youden’s index, after which the area under the ROC curve (AUC) and its 95% confidence interval (CI) were calculated. After dichotomizing the identified items at the cutoff values, odds ratios (95% CI) were obtained using multivariate logistic regression, and a diagnosis decision tree was constructed.

Finally, associations between each inflammatory biomarker and APACHE-II were examined using Pearson’s product moment correlation coefficient. All statistical analyses were performed using EZR software (version 4.1.2).

## Results

### Patient disposition and clinical characteristics

A total of 220 adult patients with radiographically confirmed pneumonia were enrolled, among whom 210 were analyzed after excluding 10 patients due to withdrawal of consent (2), diagnosis not CAP (4), only *Mycoplasma* pneumonia (3), and suspected tuberculosis (1). Regarding the causative microorganisms in the 210 patients, 15 microbial species were identified in 108 patients, whereas 9 viral species were identified in 49 patients (Table 1). Ultimately, the 210 patients were categorized into the following four groups: 77 with bacterial infection alone, 18 with viral infection alone, 31 with bacterial and viral coinfection, and 84 with no identified causative microorganism. *L. pneumophila* was classified as a bacterial species (requiring antimicrobial therapy) in this study because, compared to other atypical pneumonia caused by *M. pneumoniae* and *C. pneumoniae*, *Legionella* pneumonia presents a clinical picture more similar to bacterial pneumonia and is at risk of rapidly progressive severe pneumonia if appropriate antimicrobial therapy is not initiated promptly.

**Table 1:** Identified causative microorganisms

**Bacterial species**	**Bacterial pneumonia** **(*N* = 108)**	**Purely bacterial pneumonia** **(*N* = 77)**	**Mixed bacterial–viral pneumonia** **(*N* = 31)**
*Streptococcus pneumoniae*	48 (44.4)	29 (37.7)	19 (61.3)
*Haemophilus influenzae*	26 (24.1)	19 (24.7)	7 (22.6)
*Staphylococcus aureus*	12 (11.1)	9 (11.7)	3 (9.7)
*Klebsiella pneumoniae*	10 (9.3)	9 (11.7)	1 (3.2)
*Moraxella catarrhalis*	8 (7.4)	4 (5.2)	4 (12.9)
*Haemophilus parainfluenzae*	3 (2.8)	3 (3.9)	-
*Pseudomonas aeruginosa*	3 (2.8)	2 (2.6)	1 (3.2)
*Legionella pneumophila*	2 (1.9)	2 (2.6)	-
*Streptococcus mitis*	1 (0.9)	1 (1.3)	-
*Streptococcus pyogenes*	1 (0.9)	-	1 (3.2)
*Acinetobacter lwoffii*	1 (0.9)	1 (1.3)	-
*Capnocytophaga gingivalis*	1 (0.9)	1 (1.3)	-
*Escherichia coli*	1 (0.9)	1 (1.3)	-
*Klebsiella ornithinolytica*	1 (0.9)	1 (1.3)	-
*Klebsiella variicola*	1 (0.9)	1 (1.3)	-
**Viral species**	**Viral pneumonia** **(*N* = 49)**	**Purely viral pneumonia** **(*N* = 18)**	**Mixed bacterial–viral pneumonia** **(*N* = 31)**
Human rhinovirus/enterovirus	21 (42.9)	6 (33.3)	15 (48.4)
Human metapneumovirus	9 (18.4)	5 (27.8)	4 (12.9)
Influenza A	7 (14.3)	2 (11.1)	5 (16.1)
Respiratory syncytial virus	5 (10.2)	3 (16.7)	2 (6.5)
Parainfluenza virus 1	3 (6.1)	1 (5.6)	2 (6.5)
Coronavirus oc43	2 (4.1)	-	2 (6.5)
Parainfluenza virus 3	2 (4.1)	1 (5.6)	1 (3.2)
Adenovirus	1 (2.0)	-	1 (3.2)
Parainfluenza virus 2	1 (2.0)	1 (5.6)	-

Some patients had multiple causative pathogens

The baseline characteristics of the four groups classified according to causative pathogen are summarized in Table 2. Overall, the four groups had similar baseline characteristics for most items. However, although the distribution of CURB-65 scores was similar among the groups, the VP group had higher PSI and APACHE-II scores than other groups. The proportion of patients with chronic lung disease and purulent sputum was apparently lower in the VP group than in the other groups. None of the patients in the VP group had a sputum volume score > 2 + . In the usual practice for CAP in Japan, antigen testing for *S. pneumoniae* is more frequently performed on urine samples than on sputum specimens. The VP group had a slightly lower rate of tests aimed at detecting bacterial infection (sputum culture and urine antigen tests) and a higher rate of FilmArray. However, the implementation rate of these tests was not extremely low in the group with unknown causative microorganisms. After normalization of the analyzed variables, outliers were detected in ALP, creatinine, HbA1c, other WBC counts, and IL-8. Among these outliers, only one outlier for IL-8 (14,600 pg/mL) was excluded, with all other transformed values being used in the analysis.

**Table 2:** Baseline patient characteristics

	**Group according to causative microorganism**			
	**Purely bacterial pneumonia**	**Purely viral pneumonia**	**Mixed bacterial–viral pneumonia**	**No organism**
**Characteristic**	**(*N* = 77)**	**(*N* = 18)**	**(*N* = 31)**	**(*N* = 84)**
Male sex	44 (57.1)	10 (55.6)	18 (58.1)	45 (53.6)
Age (years), median (IQR)	74 (67–82)	68 (58–89)	75 (64–80)	74 (64–83)
Inpatient treatment	68 (88.3)	13 (72.2)	22 (71.0)	64 (76.2)
CURB-65 scores				
0	12 (15.6)	3 (16.7)	7 (22.6)	20 (23.8)
1	28 (36.4)	6 (33.3)	13 (41.9)	39 (46.4)
2	21 (27.3)	3 (16.7)	5 (16.1)	12 (14.3)
3	9 (11.7)	2 (11.1)	4 (12.9)	3 (3.6)
4	4 (5.2)	1 (5.6)	1 (3.2)	5 (6.0)
5	1 (1.3)	1 (5.6)	0 (0.0)	2 (2.4)
NA	2 (2.6)	2 (11.1)	1 (3.2)	3 (3.6)
PSI scores, median (IQR)	85 (68–105)	92 (68–127)	82 (68–111)	82 (64–102)
APACHE-II scores, median (IQR)	9 (7–12)	12 (9–14)	10 (7–13)	9 (6–12)
Underlying disease				
Congestive heart failure	10 (13.0)	3 (16.7)	2 (6.5)	9 (10.7)
Cerebrovascular disease	5 (6.5)	2 (11.1)	0 (0.0)	12 (14.3)
Dementia	9 (11.7)	4 (22.2)	2 (6.5)	5 (6.0)
Chronic lung disease	26 (33.8)	1 (5.6)	8 (25.8)	30 (35.7)
Collagen disorder	4 (5.2)	2 (11.1)	1 (3.2)	4 (4.8)
GI ulcer disease	1 (1.3)	1 (5.6)	4 (12.9)	2 (2.4)
Hepatic disease	7 (9.1)	1 (5.6)	2 (6.5)	4 (4.8)
Diabetes mellitus	12 (15.6)	4 (22.2)	7 (22.6)	21 (25.0)
Renal impairment	5 (6.5)	2 (11.1)	3 (9.7)	4 (4.8)
Acute renal failure	1 (1.3)	0 (0.0)	2 (6.5)	1 (1.2)
Hematological malignancy	1 (1.3)	0 (0.0)	1 (3.2)	1 (1.2)
Malignant tumor	7 (9.1)	1 (5.6)	2 (6.5)	9 (10.7)
Metastatic solid tumor	1 (1.3)	0 (0.0)	0 (0.0)	2 (2.4)
Sputum type				
Purulent	43 (55.8)	4 (22.2)	18 (58.1)	25 (29.8)
Mucopurulent	15 (19.5)	4 (22.2)	9 (29.0)	15 (17.9)
Mucous	14 (18.2)	5 (27.8)	4 (12.9)	23 (27.4)
NA	5 (6.5)	5 (27.8)	0 (0.0)	21 (25.0)
Sputum-volume score				
0 (none)	6 (7.8)	7 (38.9)	1 (3.2)	24 (28.6)
1 + (< 10 mL/day)	38 (49.4)	8 (44.4)	10 (32.3)	35 (41.7)
2 + (10–49 mL/day)	18 (23.4)	3 (16.7)	12 (38.7)	19 (22.6)
3 + (50–99 mL/day)	12 (15.6)	0 (0.0)	7 (22.6)	5 (6.0)
4 + (> 100 mL/day)	3 (3.9)	0 (0.0)	1 (3.2)	1 (1.2)
Identifying method of causative microorganism				
Sputum culture	74 (96.1)	15 (83.3)	30 (96.8)	66 (78.6)
*S. pneumoniae* SAT	27 (35.1)	3 (16.7)	7 (22.6)	25 (29.8)
*S. pneumoniae* UAT	73 (94.8)	16 (88.9)	29 (93.5)	79 (94.0)
*Legionella* UAT	71 (92.2)	15 (83.3)	29 (93.5)	77 (91.7)
FilmArray	63 (81.8)	18 (100.0)	31 (100.0)	71 (84.5)

Values are presented as n (%), unless otherwise indicated

*APACHE-II* Acute Physiology and Chronic Health Evaluation II, *CURB-65* Confusion, Urea, Respiratory Rate, Blood Pressure and Age ≥ 65 Years, *GI* gastrointestinal, *IL* interleukin, *IQR* interquartile range, *NA* not available, *PSI* pneumonia severity index, *SAT* sputum antigen test, *UAT* urinary antigen test

### Predictors for the diagnosis of BP

The BP group comprised patients with bacterial infection alone and bacterial and viral coinfection (*N* = 108). Patients with bacterial and viral coinfections were classified into the BP group given that they required antimicrobial therapy. This group was then compared to the VP group. The baseline characteristics of the two groups are presented in Table 3. Notably, no significant differences in sex, age, or disease severity were observed between two groups. Among inflammation-related items, neutrophil count, sputum volume, sputum type, IL-8, IL-10, and AAT showed a *p* value of < 0.05 during univariate or multivariate logistic analyses. No significant differences in the other items were observed. Levels of CRP, IL-6, IL-8, IL-10, procalcitonin, presepsin, and pentraxin 3 showed a significant correlation with APACHE-II score. However, AAT showed no correlation (Table 4).

**Table 3:** Comparison of patient baseline characteristics between the bacterial pneumonia (BP) and purely viral pneumonia (VP) groups

**Characteristic**	**BP group**^**a**^	**VP group**	**Univariate**^**b**^	**Multivariate**^**c**^
	**(*N* = 108)**	**(*N* = 18)**	***p*-value**	***p*-value**
Male sex, n (%)	62 (57.4)	10 (55.6)	1	
Age (years)	75 (66–82)	68 (58–89)	0.786	
APACHE-II scores	9 (7–12)	12 (9–14)	0.091	
PSI scores	84 (68–109)	92 (68–127)	0.551	
Sputum type, n (%)				
P/PM	85 (78.7)	8 (44.4)	0.007	0.004
M/blank	23 (21.3)	10 (55.6)		
Sputum-volume scores, n (%)				
0	7 (6.5)	7 (38.9)	< 0.001	< 0.001
1 +	48 (44.4)	8 (44.4)		
2 +	30 (27.8)	3 (16.7)		
3 +	19 (17.6)	0 (0.0)		
4 +	4 (3.7)	0 (0.0)		
Neutrophils (cells/µL)	9710 (7428–12,465)	6780 (4737–11,100)	0.039	0.038
Lymphocytes (cells/µL)	1072 (770–1565)	924 (667–1325)	0.356	0.360
Platelets (10^4^ cells/µL)	22 (16–28)	19 (16–23)	0.200	0.279
C-reactive protein (mg/L)	123 (71–192)	90 (32–120)	0.055	0.096
IL-6 (pg/mL)	104 (44–234)	62 (42–249)	0.481	0.500
IL-8 (pg/mL) ^d^	8 (8–13)	13 (8–21)	0.033	0.018
IL-10 (pg/mL)	1.2 (0.8–3.1)	5.4 (3.5–8.8)	< 0.001	< 0.001
α1-antitrypsin (mg/dL)	222 (189–266)	186 (159–199)	< 0.001	0.002
Procalcitonin (ng/mL)	0.21 (0.08–0.61)	0.14 (0.07–0.28)	0.345	0.420
Presepsin (pg/mL)	241 (156–409)	389 (196–524)	0.211	0.242
Pentraxin 3 (ng/mL)	9 (5–20)	17 (11–28)	0.196	0.323
Random plasma glucose (mg/dL)	123 (109–155)	128 (116–153)	0.461	0.411
Hemoglobin A1c (%)	5.8 (5.6–6.4)	5.9 (5.7–6.4)	0.858	0.569
Aspartate aminotransferase (IU/L)	24 (18–33)	31 (23–54)	0.201	0.371
Alanine aminotransferase (IU/L)	19 (13–29)	18 (12–43)	0.960	0.697
Alkaline phosphatase (IU/L)	243 (198–314)	243 (180–316)	0.841	0.848
γ-glutamyl transpeptidase (IU/L)	28 (18–49)	43 (20–69)	0.198	0.112
Lactate dehydrogenase (IU/L)	214 (180–262)	233 (178–275)	0.571	0.519
Total bilirubin (mg/dL)	0.80 (0.50–1.08)	0.65 (0.4–1.00)	0.071	0.068
Blood urea nitrogen (mg/dL)	17 (12–23)	17 (11–26)	0.911	0.742
Creatinine (mg/dL)	0.83 (0.67–1.04)	0.95 (0.78–1.01)	0.270	0.354

Values are presented as median (interquartile range), unless otherwise indicated

*APACHE-II* Acute Physiology and Chronic Health Evaluation II, *IL* interleukin, *M* mucous, *PSI* pneumonia severity index, *P* purulent, *PM* purulent-mucous

^a^BP group consisted of patients with purely bacterial pneumonia and mixed bacterial–viral pneumonia

^b^Continuous variables were compared using Mann–Whitney U test, whereas binary variables were compared employing Fisher’s exact test

^c^Each variable was evaluated using multivariate logistic regression analysis adjusting for sex and age

^d^Detection limit for IL-8 was 8 pg/mL

**Table 4:** Pearson correlation between APACHE-II score and biomarkers at diagnosis

	**n**	**Coefficient, r**	**95% CI**	***p*-value**
CRP	209	0.136	0.0005–0.267	0.049
IL-6	210	0.442	0.327–0.545	< 0.001
IL-8	208	0.393	0.271–0.502	< 0.001
IL-10	210	0.366	0.243–0.478	< 0.001
α1-antitrypsin	210	0.019	− 0.117–0.154	0.786
Procalcitonin	210	0.366	0.243–0.478	< 0.001
Presepsin	209	0.403	0.283–0.511	< 0.001
Pentraxin 3	209	0.376	0.253–0.487	< 0.001

*APACHE-II* Acute Physiology and Chronic Health Evaluation II, *CI* confidence interval, *IL* interleukin

**Fig. 1:**
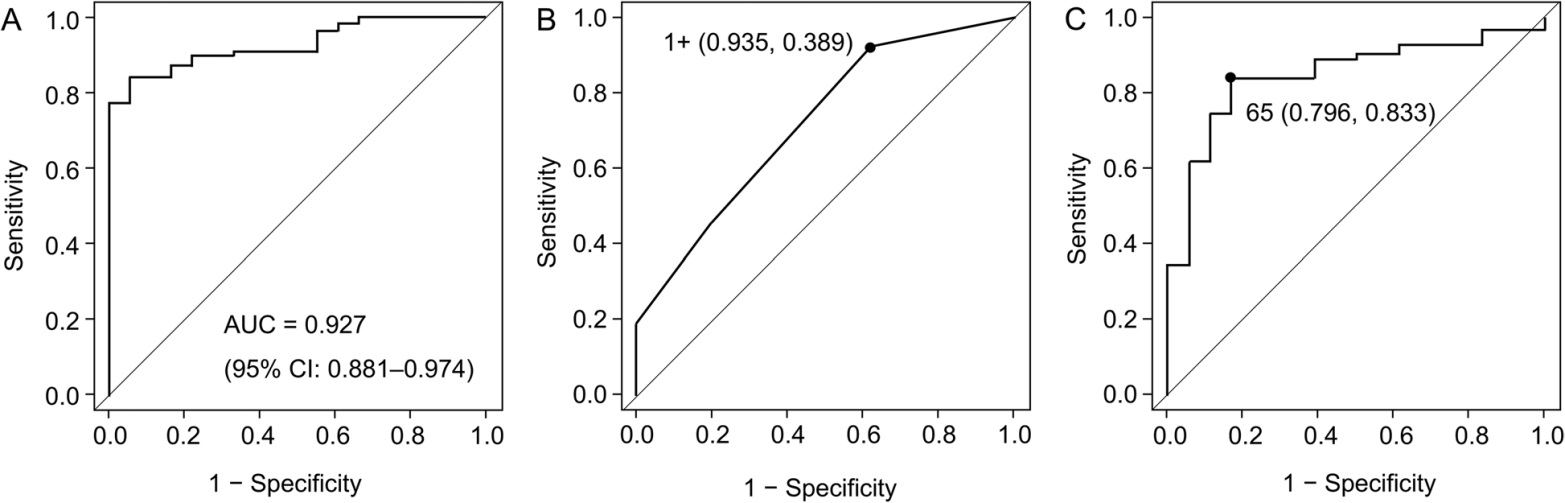
Receiver operating characteristic curves for bacterial pneumonia in 126 patients with community-acquired pneumonia. **A** Sputum volume, IL-10, and AAT. **B** Sputum-volume score. **C** α1-antitrypsin/IL-10 ratio. The values in Figures (**B**) and (**C**) are cutoff values (sensitivity and specificity). AUC: the area under the curve; CI: confidence interval

Repeat logistic regression analysis using selected inflammation-related markers, age, and sex showed that sputum volume, IL-10, and AAT significantly differentiated between the BP and VP groups (Table 5). The results suggested that a large sputum volume, a higher AAT value, and a lower IL-10 value were significant predictors of bacterial infection. Moreover, the ROC curve for the combination of these three variables showed an AUC of 0.927 (95% CI: 0.881–0.974) (Fig. 1A).

**Table 5:** First, intermediate, and final logistic regression results for factors differentiating between bacterial pneumonia and purely viral pneumonia

	**Results**		
	**First**	**Intermediate**	**Final**
**Variable**	***p*-value**	***p*-value**	***p*-value**
Sex	0.920	0.457	
Age	0.574	0.663	
Sputum type	0.842		
Sputum-volume score	0.003	< 0.001	< 0.001
Neutrophils	0.075		
IL-8	0.606		
IL-10	0.002	< 0.001	< 0.001
α1-antitrypsin	0.018	0.007	0.008

*IL* interleukin

For easier use of the variables, cutoff values were obtained using the ROC curves. The ROC curve for sputum volume score showed an AUC of 0.752 (95% CI: 0.642–0.862) and a maximum Youden’s index of 1.324 with a sensitivity and specificity of 0.935 and 0.389, respectively, corresponding to a diagnostic cutoff of 1 + (Fig. 1B). The ROC curve for the AAT showed an AUC of 0.748 (95% CI: 0.647–0.850) and a maximum Youden’s index of 1.389 with a sensitivity and specificity of 0.611 and 0.788, respectively, corresponding to a diagnostic cutoff of 204. The ROC curve for IL-10 showed an AUC of 0.798 (95% CI: 0.697–0.899) and a maximum Youden’s index of 1.602 with a sensitivity and specificity of 0.769 and 0.833, respectively, corresponding to a diagnostic cutoff of 3.14. When AAT and IL-10 were analyzed separately, the BP group had higher levels of AAT and lower levels of IL-10 than the VP group. In other words, AAT and IL-10 were inversely correlated in terms of predicting bacterial infection. Therefore, we propose to use the AAT/IL-10 ratio with a single cutoff value. The ROC curve for the AAT/IL-10 ratio showed an AUC of 0.824 (95% CI: 0.736–0.913) and a maximum Youden’s index of 1.630 with a sensitivity and specificity of 0.796 and 0.833, respectively, corresponding to a diagnostic cutoff of 65 (Fig. 1C). The results indicated that AAT/IL-10 ratio was more discriminative of bacterial infection than AAT and IL-10 alone. Logistic regression analysis using dichotomized variables at the cutoff values showed that the odds ratios for the diagnosis of bacterial pneumonia were 10.4 for sputum volume score and 19.8 for AAT/IL-10 ratio (Table 6).

**Table 6:** Odds ratios for the definitive diagnosis of bacterial pneumonia by biomarkers

Variable	Odds ratio	95% CI	*p*-value
Sputum volume (absence vs. presence)	10.4	2.2–50.2	0.004
α1-antitrypsin/IL-10 ratio (< 65 vs. ≥ 65)	19.8	4.7–83.2	< 0.001

*CI* confidence interval

### Diagnostic decision tree

**Fig. 2:**
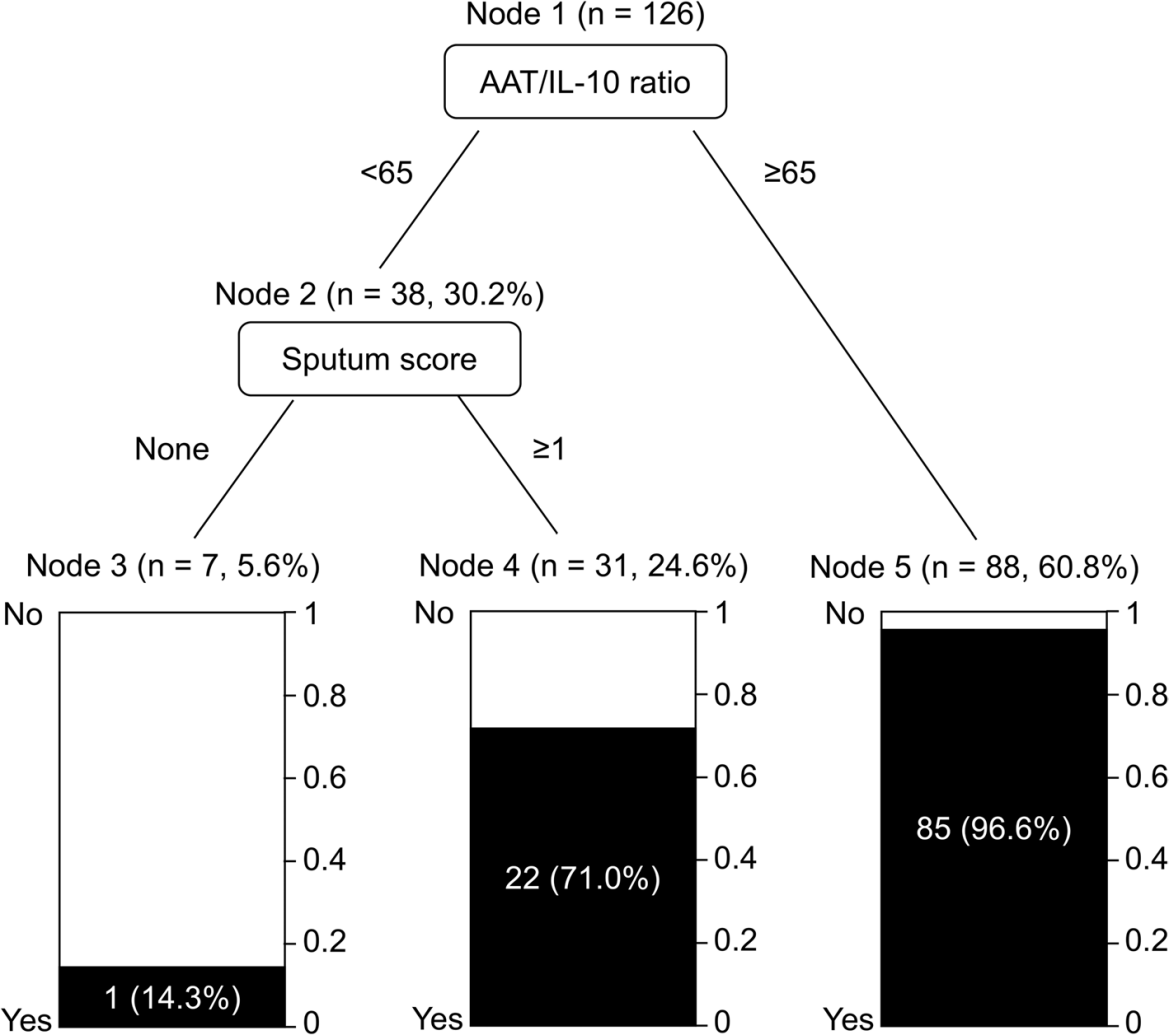
Diagnostic decision tree of bacterial pneumonia in patients with community-acquired pneumonia. Black and white zones represent bacterial and purely viral pneumonia, respectively

A diagnostic decision tree for bacterial pneumonia was constructed using the two predictors (Fig. 2). Accordingly, almost all of the patients with an AAT/IL-10 ratio of ≥ 65 had bacterial pneumonia. Meanwhile, most of the patients with an AAT/IL-10 ratio of < 65 and less sputum had purely viral pneumonia. We also performed the decision tree analysis for 84 patients in which no causative organism was identified (Fig. S2). The distribution pattern of patients was similar to the results obtained in the patient group in which the causative microorganism was identified.

## Discussion

Previous reports have shown that the detection rate of causative microorganisms in patients with CAP is approximately 40% [11, 12]. In this multicenter prospective study, all participating centers, with infectious disease specialists serving as principal investigators, performed rigorous microbiological testing on all enrolled patients and were able to identify the causative microorganism in 60% of cases. The most common type of pneumonia was bacterial pneumonia (36%), among which the majority was caused by *S. pneumoniae* and *Haemophilus influenzae*. This was followed by bacterial and viral coinfection (15%) and purely viral infection (9%). This distribution closely resembles that observed by studies conducted in Europe [12, 20, 21]. Because bacterial and viral coinfection is more common than viral infection alone in CAP, detecting a virus using polymerase chain reaction does not imply that empiric therapy using antibiotics is unnecessary. Therefore, more objective evidence suggesting that patients require antimicrobials is important. In general, patients with purely viral pneumonia present with dry cough without sputum production, whereas those with bacterial pneumonia often present with purulent sputum and an increased WBCs count with neutrophil predominance [22, 23]. However, because these clinical symptoms and findings alone do not clearly distinguish bacterial pneumonia from purely viral pneumonia, we attempted to improve the prediction accuracy by combining biomarkers as an auxiliary diagnosis. After exploring variables obtained through laboratory investigations, including sputum volume, sputum type, WBC count, and biomarkers, we found that sputum volume, IL-10, and AAT levels were useful to differentiate between the BP and VP groups. Purulent sputum, increase in neutrophil count, and low IL-8 value may also be weak predictors of bacterial pneumonia; however, procalcitonin, presepsin, pentraxin 3, and IL-6 were not associated with etiology of CAP.

Among the cytokines examined, only IL-10 alone was able to differentiate BP from VP. IL-10 suppresses inflammation signaling to T cells and macrophages, whereas IL-6 and IL-8 promotes inflammation by activating B cells, T cells, and neutrophils. Our results suggest IL-10 does not respond to bacterial infection and remains below abnormal levels (5 pg/mL) in the early stage of bacterial pneumonia. In contrast, IL-10 elevation was observed in the VP group. A similar elevation in IL-10 was reported in pneumonia caused by influenza viruses [24] and COVID-19 [25]. The levels of IL-6 and IL-8 were similarly increased in the BP and VP groups in the present study.

A previous study reported that serum concentrations of AAT at the time of admission were closely correlated to in-hospital morbidity and fever duration in patients with CAP requiring hospitalization [26]. Conversely, patients with severe COVID-19 have been reported to have lower AAT levels than patients hospitalized due to non-COVID-19 pneumonia [27]. In the present study, AAT level was not associated with disease severity and was significantly more increased in the BP group than in the VP group. These results suggest that AAT is a protein that preferably responds to bacterial infection. As mentioned above, since IL-10 and AAT change in contrasting directions, we propose that an AAT/IL-10 ratio of 65 or more is a good predictor of bacterial pneumonia, regardless of disease severity at diagnosis.

For people presenting with symptoms of lower respiratory tract infection in primary care, the NICE guideline recommends using the results of CRP to guide the prescription of antibiotics if a diagnosis of pneumonia has not been made [28], while all patients with CAP should be offered an antibiotic [29]. In our study population, the median CRP values for the BP and VP groups were 123 mg/L (interquartile range [IQR]: 71–192) and 90 (IQR: 32–120) mg/L, respectively, but the difference was not statistically significant. It is important to note that CRP is a useful biomarker to guide general practitioners’ decisions about antibiotic prescription only when used in the right population [30]. Several studies have shown that procalcitonin could be a useful biomarker in differentiating between bacterial and viral infections [5, 31, 32] considering that its serum levels tend to be higher in bacterial infections. However, no clear cutoff value has yet been established [33]. Furthermore, reports reveal that procalcitonin showed a wide sensitivity range in the detection of bacterial infection [34]. Procalcitonin levels are also unlikely to provide reliable evidence to require the administration of antibiotics or enable withholding such treatment in patients with CAP [35]. For such reasons, the ATS/IDSA guideline does not recommend the use of procalcitonin to guide decisions regarding the initiation of antimicrobial drugs (bacterial or viral differentiation) [36]. Moreover, procalcitonin-guided antibiotic treatment does not promote lower antimicrobial use compared to routine care in patients who presented to the emergency department with a suspected lower respiratory tract infection [6]. In fact, procalcitonin has been significantly associated with severity of illness, similar to presepsin [37–40], consistent with the findings in the current study. Therefore, procalcitonin may be more appropriately used as a marker of sepsis.

Establishing a definitive etiological diagnosis is difficult and time consuming. Therefore, a decision tree using sputum volume, IL-10, and AAT can be useful in predicting bacterial pneumonia and promoting the appropriate use of antibiotics, even when airway specimens are not easily collected, as in the case of the COVID-19 pandemic. Biomarkers are a simple and rapid supplementary diagnostic tool that can be used in various situations. However, biomarker measurements are too costly to incorporate into routine care. Therefore, continuous research and development of diagnostic systems that could more efficiently detect causative pathogens from patients with pneumonia is warranted.

### Limitations

This study has some limitations worth elaborating. The number of patients included for analysis was quite small, especially for those classified as the VP group, leading to only 6 events per variable (EPV). However, given the exploratory nature of this study, the results for a model with 5–9 EPV appear to be acceptable [41]. Disease severity was not considered in the regression analyses, although some variables were correlated with severity. However, no significant difference in APACHE-II score or PSI was observed between the BP and VP groups.

COVID-19 cases were excluded from the present study at the discretion of their attending physicians despite not being explicitly stated in the exclusion criteria. When the study was first planned, we had not anticipated a coronavirus outbreak during the study period. At that time, sputum collection and examination were difficult because of  insufficient information on SARS-CoV-2 and lack of clear infection control measures. The FilmArray respiratory panel can detect a limited number of virus types, although the main viruses were covered. As such, the assay using samples obtained at the upper respiratory tract may be only an estimate of the presence of virus in that area. However, in previous studies that evaluated the diagnostic performance of the FilmArray respiratory panel for viral CAP, viruses detected in nasopharyngeal swab specimens were highly consistent with the results of viral culture and genetic testing of lower respiratory tract specimens obtained by bronchoscopy [42–44]. Other limitations include the lack of data regarding changes in biomarkers throughout the clinical course of pneumonia and the lack of a validation cohort. It is important to note that the predictors of bacterial infection found in this study were derived from a group of patients in whom causative microorganism(s) were identified, and it is unknown at this stage whether these factors can be accurate indicators in a group of patients in whom no causative microorganism was identified. In addition, all patients enrolled in the study were attempted to submit to a sputum examination, and sputum culture results were the primary diagnostic basis for bacterial infection. Since bronchoscopy was not performed on all patients, it was difficult to prove whether patients who did not produce sputum really did not have bacterial infection. Collectively, our findings should be validated by further robust studies.

## Conclusions

Considering that obtaining a definitive diagnosis with the current testing methods is difficult and time consuming, a decision tree with two predictors, namely sputum volume and the AAT/IL-10 ratio, can be useful in predicting bacterial pneumonia among patients diagnosed with CAP and facilitating the appropriate use of antibiotics.

### Supplementary Information

**Additional file 1: Fig. S1.** Flow diagram for the classification of pneumonia. **Fig. S2**. Diagnostic decision tree of bacterial pneumonia in patients with community acquired pneumonia in which no causative organism was identified. **Appendix.** Collaborators.

## Data Availability

The anonymized datasets for analysis of the present study are available from the corresponding author on reasonable request.

## Abbreviations

AAT: α1-Antitrypsin
ALP: Alkaline phosphatase
APACHE-II: Acute Physiology and Chronic Health Evaluation II
BP: Bacterial pneumonia
CAP: Community-acquired pneumonia
CI: Confidence interval
COVID-19: Coronavirus disease 2019
CRP: C-reactive protein
HbA1c: Hemoglobin A1c
IL: Interleukin
CURB-65: Confusion, Urea, Respiratory Rate, Blood Pressure and Age ≥ 65 Years
PSI: Pneumonia Severity Index
VP: Purely viral pneumonia
WBC: White blood cell
