# Supplementary material for: A high α1-antitrypsin/interleukin-10 ratio predicts bacterial pneumonia in adults with community-acquired pneumonia: a prospective cohort study
Source: Pneumonia (Nathan). 2023 Oct 25;15:16. doi: 10.1186/s41479-023-00118-4 (PMC10599029; doi:10.1186/s41479-023-00118-4)
Supplement: Supplementary file 1 — Additional file 1: Fig. S1. Flow diagram for the classification of pneumonia. Fig. S2. Diagnostic decision tree of bacterial pneumonia in patients with community acquired pneumonia in which no causative organism was identified. Appendix. Collaborators. [file 41479_2023_118_MOESM1_ESM.pdf]

A high α1-antitrypsin/interleukin-10 ratio predicts bacterial pneumonia in adults with community-acquired pneumonia: A prospective cohort study

Miyazaki T, et al.

Supplemental Material

Supplementary Figures

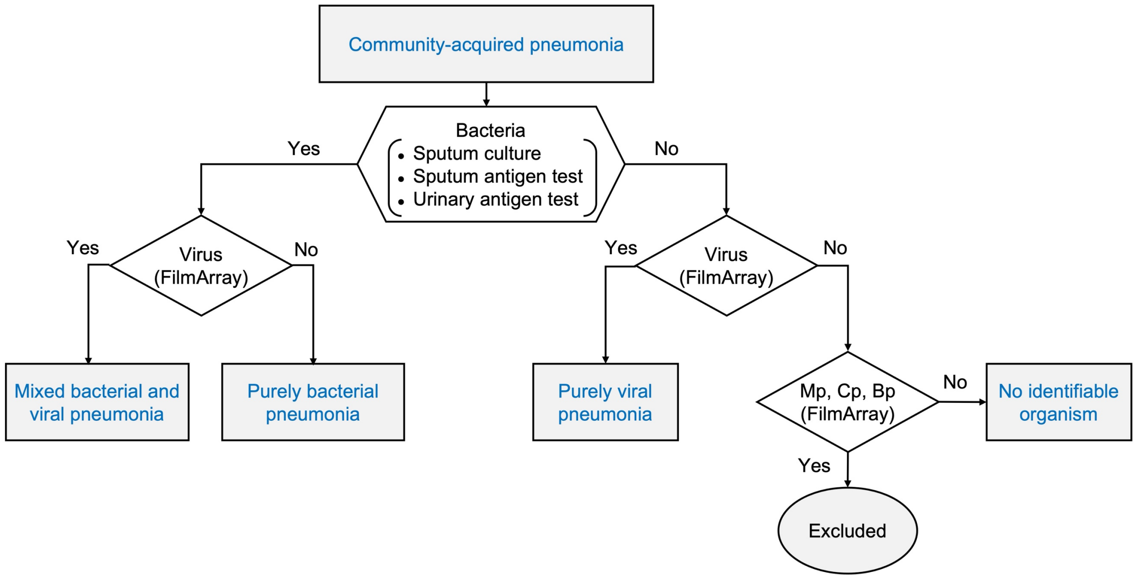

Fig. S1 Flow diagram for the classification of pneumonia

Mp: *Mycoplasma pneumoniae*; Cp: *Chlamydophila pneumoniae*; Bp: *Bordetella pertussis*

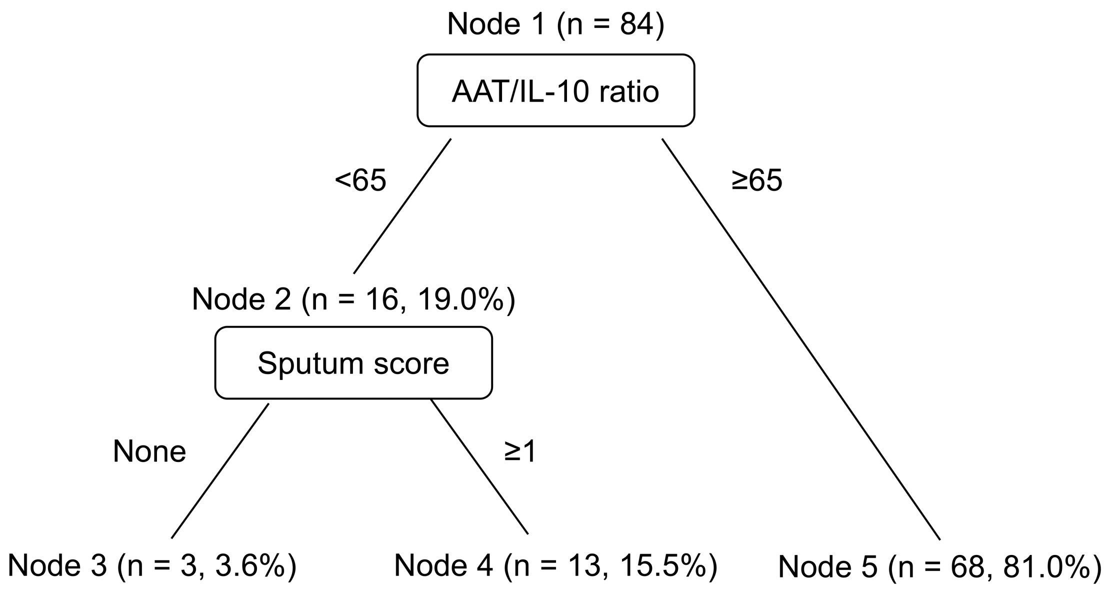

Fig. S2 Diagnostic decision tree of bacterial pneumonia in patients with community-acquired pneumonia in which no causative organism was identified.

(Appendix) Collaborators

Nagasaki University Hospital, Nagasaki, Japan

Fumiko Hayashi, Yuya Ito, Satoshi Irifune

Japanese Red Cross Nagasaki Genbaku Isahaya Hospital, Iasahaya, Japan

Akira Kondo, Kazumasa Akagi, Naomi Ehara, Ritsuko Miyashita, Toyoshi Matsutake

Japanese Red Cross Nagasaki Genbaku Hospital, Nagasaki, Japan

Takumi Nakao, Yosuke Harada, Noritaka Honda, Ryuta Tagawa, Yohei Koide, Takeshi Kitazaki

Isahaya General Hospital, Isahaya, Japan

Shinya Tomari, Sakiko Tanaka-Moriyama

Sasebo Chuo Hospital, Sasebo, Japan

Takashi Kitamikado

Nagasaki Harbor Medical Center, Nagasaki, Japan

Yosuke Harada, Mutsumi Ozasa, Sumako Yoshioka, Ryosuke Morio, Shotaro Ide, Kazumasa Akagi

University of Occupational and Environmental Health, Japan, Kitakyushu, Japan

Toshiki Morimoto, Hiroaki Ikegami, Takashi Tachiwada, Hideki Kanda, Sachika Hara, Yuto Iwanaga, Yasuhiko Nikaido, Toshinori Kawanami, kei Yamasaki, Taiki Manabe

Kouseikai Hospital, Nagasaki, Japan

Yohsuke Nagayoshi, Toyoshi Matsutake, Nobuo Morikawa

Sasebo City General Hospital, Sasebo, Japan

Yasuhiro Tanaka, Midori Shimada, Masataka Yoshida, Tatsuhiko Harada, Asuka Umemura

Saiseikai Nagasaki Hospital, Nagasaki, Japan

Nana Nakada, Keiko Iida

Aino Memorial Hospital, Unzen, Japan

Yuichi Inoue, Shintaro Hara

Nagasaki Medical Center, Omura, Japan

Kazuaki Takeda
